# Molecular Survey of Rodent-Borne Infectious Agents in the Ferlo Region, Senegal

**DOI:** 10.3390/genes14051107

**Published:** 2023-05-18

**Authors:** Joa Braïthe Mangombi-Pambou, Laurent Granjon, Fabien Flirden, Mamadou Kane, Youssoupha Niang, Bernard Davoust, Florence Fenollar, Oleg Mediannikov

**Affiliations:** 1 Centre Interdisciplinaire de Recherches Médicales de Franceville (CIRMF), Franceville BP 769, Gabon; joa.mangombi@gmail.com; 2 Department of Epidemiology of Parasitic Diseases, Aix-Marseille University, IRD, AP-HM, MEPHI, 13005 Marseille, France; fabienflirden@gmail.com (F.F.); bernard.davoust@gmail.com (B.D.); 3 IHU Méditerranée Infection, 13005 Marseille, France; florence.fenollar@univ-amu.fr; 4 CBGP, IRD, CIRAD, INRAE, Institut Agro, University Montpellier, 34000 Montpellier, France; laurent.granjon@ird.fr; 5 BIOPASS, CBGP-IRD, ISRA, UCAD, CIRAD, Campus de Bel-Air, Dakar 1386, Senegal; mamadou.kane@ird.fr (M.K.);; 6 IRD, AP-HM, SSA, VITROME, Aix-Marseille University, 13005 Marseille, France

**Keywords:** rodents, infectious agents, multiple infections, zoonotic diseases, tick-borne zoonotic disease

## Abstract

Zoonotic pathogens are responsible for most infectious diseases in humans, with rodents being important reservoir hosts for many of these microorganisms. Rodents, thus, pose a significant threat to public health. Previous studies in Senegal have shown that rodents harbour a diversity of microorganisms, including human pathogens. Our study aimed to monitor the prevalence of infectious agents in outdoor rodents, which can be the cause of epidemics. We screened 125 rodents (both native and expanding) from the Ferlo region, around Widou Thiengoly, for different microorganisms. Analysis, performed on rodent spleens, detected bacteria from the *Anaplasmataceae* family (20%), *Borrelia* spp. (10%), *Bartonella* spp. (24%) and *Piroplasmida* (2.4%). Prevalences were similar between native and the expanding (*Gerbillus nigeriae*) species, which has recently colonised the region. We identified *Borrelia crocidurae*, the agent responsible for tick-borne relapsing fever, which is endemic in Senegal. We also identified two other not-yet-described bacteria of the genera *Bartonella* and *Ehrlichia* that were previously reported in Senegalese rodents. Additionally, we found a potential new species, provisionally referred to here as *Candidatus* Anaplasma ferloense. This study highlights the diversity of infectious agents circulating in rodent populations and the importance of describing potential new species and evaluating their pathogenicity and zoonotic potential.

## 1. Introduction

*Rodentia* is the most successful and diversified order of living mammals, representing about 40% of all mammalian species [1]. They have a very broad ecological spectrum, and their presence in different types of biotopes allows some species to live in close proximity to wildlife, livestock and humans [2,3]. Rodents are well known as important reservoirs of infectious agents, which they can transmit to humans [4]. They are the source of many zoonotic pathogens, including *Borrelia* spp., *Leptospira* spp., *Bartonella* spp. and *Trypanosoma* spp. [4,5].

Several rodent-borne zoonotic infectious agents and their associated diseases circulate in Africa. These include plague [6,7], Lassa haemorrhagic fever [8,9] and leptospirosis [10]. Many studies have described the presence not only of known zoonotic pathogens in rodents but also of microorganisms the pathogenicity and zoonotic potential of which are not yet known [11,12,13,14,15].

In Senegal, many studies have focused on small mammals, especially rodents, that inhabit the country, where they can represent reservoirs of infectious and potentially zoonotic agents [3,16]. This is the case in the Ferlo region of northern Senegal and has become evident, in particular, as part of a major multidisciplinary project connected with the pan-African Great Green Wall initiative, aimed at mitigating the effects of desertification and environmental degradation in the Sahelian environment: “https://ohmi-tessekere.in2p3.fr/ (accessed on 25 February 2023)”.

One of these studies detected the presence of several known zoonotic infectious agents (bacteria and parasites) and described new infectious agents in rodents from indoor and outdoor habitats in the Ferlo region [12]. This study concerned, among others, the Nigerian gerbil *G. nigeriae*, a species that has recently and rapidly colonised northern Senegal, and the domestic mouse *Mus musculus* sp.), an exotic invasive species currently expanding in most of the country [17,18,19,20]. Another study recently highlighted an epizootic outbreak of Q fever in rodents from the Ferlo region [21]. This outbreak was caused by a new genotype of *Coxiella burnetii*, the pathogenicity of which is still unknown. It was recent and is probably ongoing since previous studies of rodents from the same region revealed no evidence of the Q fever agent [12]. This study [21] contributes towards the same subject matter and was published separately and urgently due to the importance of the results.

These data show the importance of monitoring zoonotic pathogens potentially transmitted by rodents. Indeed, rodents are sentinels of infectious diseases that can allow the early detection and management of zoonoses [22]. This is the context in which we carried out this molecular epidemiological survey of microorganisms carried by rodent populations in Ferlo in order to address the public health risks for human populations in contact with these small mammals.

## 2. Material and Methods

### 2.1. Study Area and Sample Design

**Figure 1:**
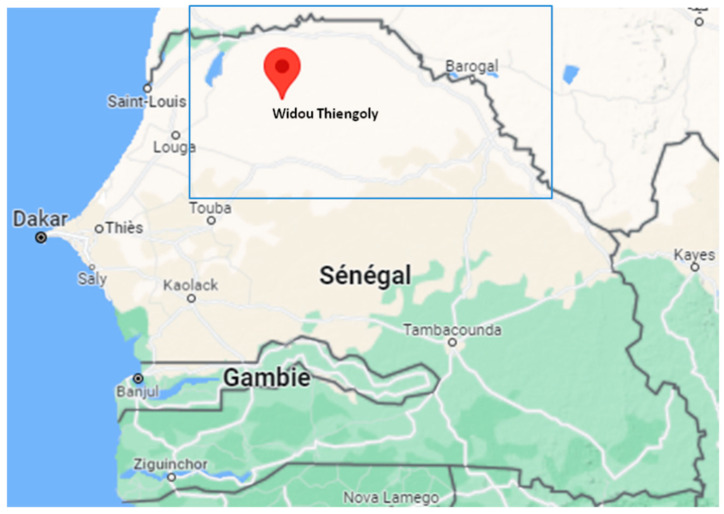
Map of the location of Widou Thiengoly, the area where rodents were collected in the Ferlo region (in the blue box), northern Senegal.

Rodent sampling was conducted in the Ferlo region over two sampling periods in outdoor habitats around three temporary ponds east of Widou Thiengoly (average coordinates 15.96° N; 15.25° W), as shown in Figure 1. The first took place at the end of the 2019 rainy season (September–October 2019) and the second at the end of the 2020 dry season (June–July 2020). The trapping methodology was described elsewhere [19], with traps being placed either in lines of 20 to 40 traps spaced 10 m apart or opportunistically, depending on signs of the presence of rodents. In addition, “night hunts” were carried out at each site to catch species that are difficult to capture by hand (small Gerbillinae, jerboas, etc.). All the individuals were euthanatized by cervical dislocation, then autopsied, and samples (spleen in 95% ethanol) were extracted from each in order to search for the parasites and pathogens they harboured.

### 2.2. Ethical Statement Regarding Fieldwork 

The fieldwork was conducted within the framework of agreements between the Institut National de Recherche pour le Développement (IRD) and the Republic of Senegal, as well as with the Senegalese Water and Forest Management Head Office of the Ministry of Environment and Sustainable Development. None of the rodent species investigated in this study have a protected status (see IUCN and CITES lists). Handling procedures were carried out under the CBGP agreement for experiments on wild animals (no. D-34-169-1) and followed the official guidelines of the American Society of Mammalogists [23]. The trapping campaigns were carried out with the explicit prior agreement of the competent local authorities.

### 2.3. DNA Extraction

DNA extraction was performed on a BioRobot EZ1 (Qiagen, Courtaboeuf, France) using a commercial EZ1 DNA Tissue Kit (Qiagen, Hilden, Germany) according to the manufacturer’s instructions. Total DNA was extracted from 10 mg of spleen from all rodent samples that were preserved in alcohol. DNA was eluted in 100 μL of TE buffer and stored at −20 °C until used for PCR amplification. 

### 2.4. PCR Amplification

Real-time PCR (qPCR) was performed to screen all rodent samples using previously reported primers and probes [12] for *Bartonella* spp., *Anaplasmataceae*, *Borrelia* spp., *Rickettsia* spp., *Piroplasmida*, *Mycoplasma* spp., pan-*Filaria*, pan-*Kinetolastidae* and pan-*Leishmania–Trypanosoma*.

For each qPCR run, the final volume of 20 μL was composed of 10 μL of the Roche master mix (Roche Applied Science, Mannheim, Germany), 3 μL of water, 0.5 μL of each primer (20 μM) and probe (5 μM), 0.5 μL of UDG (uracil DNA glycosylase) and 5 μL of DNA extracted from the spleen. Amplification was performed in a CFX96 Real-Time PCR detection system (Bio-Rad Laboratories, Foster City, CA, USA) according to the following thermal profile: one step at 50 °C for 2 min for UDG action (eliminating PCR amplicon contaminants), an initial denaturation at 95 °C for 5 min, followed by 40 cycles at 95 °C for 15 s and 60 °C for 30 s for annealing extension. For all systems, any sample with a cycle threshold (Ct) value of less than 38 Ct was considered positive. The sequences of the primers and probes are shown in Table 1. 

Conventional PCR analysis was performed in an automated DNA thermal cycler (GeneAmp PCR Systems Applied Biosystems, Courtaboeuf, France) for all qPCR-positive samples using the primers and conditions described in Table 1. The amplification reaction was conducted in a final volume of 25 μL containing 12.5 μL of Ampli Taq Gold master mix, 0.75 μL of each primer (10 μM), 5 μL of DNA template and 6 μL of water. The thermal cycling profile consisted of one incubation step at 95 °C for 5 min, 45 cycles of 30 s at 95 °C, 30 s to 1 min at the annealing temperature (Table 1) and 1 min at 72 °C, and a final extension step of 5 min at 72 °C. Successful amplification was confirmed by electrophoresis in a 1.5% agarose gel, and the amplicons were completely sequenced on both strands.

For each assay, DNA extracts of the targeted bacteria or parasites (laboratory colony) were used as positive controls and distilled water as the negative control (Table S1).

### 2.5. Sequencing and Phylogenetic Analysis

Sequencing analyses were performed on an ABI Prism 3130XL Genetic Analyzer (Applied Biosystems, Thermo Fisher Scientific, Illkirch-Graffenstaden, France) using a DNA sequencing BigDye Terminator V3.1 Cycle Sequencing Kit (Applied Biosystems, Foster City, CA, USA, PerkinElmer) according to the manufacturer’s instructions. The BigDye products were purified on Sefadex G-50 Superfine gel filtration resin (Cytiva, Formerly GE Healthcare Life Science, Lund, Sweden). The obtained sequences were analysed using ChromasPro version 1.3 (Technelysium Pty, Ltd., Tewantin, QLD, Australia) for assembly and were aligned with other sequences of targeted bacteria or parasite species from GenBank using CLUSTALW, implemented in BioEdit v7.2 [33]. Phylogenetic trees were constructed with MEGA software v.7 [34]. The Maximum Likelihood method based on the Hasegawa–Kishino–Yano model (HKY) was used to infer the phylogenetic analysis with 500 bootstrap replicates.

### 2.6. Statistical Analysis 

Statistical analysis was performed with R software V4.1.2 [35] using Chi-square/Fisher’s exact tests or Wilcoxon–Mann–Whitney for data comparisons between the prevalence of infected rodents for all parasites according to their sex, age class (distinguished using the weight criteria provided in [3]), status (expanding *G. nigeriae* or native) and captured season. When *p*-values were <0.05, they were considered to be significant.

## 3. Results

### 3.1. Samples Included in the Study

A total of 125 small mammals captured in the Ferlo region were analysed in this study. The animals captured included the expanding species *G. nigeriae* (71/125, 56.8%), which was the most abundant species, and six native species, namely, *Arvicanthis niloticus* (29/125, 23.2%), *Desmodilliscus braueri* (3/125, 2.4%), *Gerbillus nancillus* (9/125, 7.2%), *Jaculus jaculus* (4/125, 3.2%), *Taterillus* sp. (corresponding most probably to *Taterillus pygargus*; 8/125, 6.4%) and *Xerus erythropus* (1/125, 0.8%) (Table 2).

### 3.2. Molecular Detection of Microorganisms (Bacteria and Protozoa)

All rodents were found to be negative by qPCR for pan-*Kinetoplastidae*, pan-*Leishmania–Trypanosoma*, *Rickettsia* spp., *Mycoplasma* spp. And pan-*Filaria*. We also included here the results on *C. burnetii*, which were published separately, because these results originated from the same rodents. Thus, 67/125 (53.6%) rodents were positive for at least one of the five other qPCR screening systems (Table 2). The most common microorganism detected was *Bartonella* spp. at 24% (30/125) followed by *C. burnetii* at 22.4% (28/125), *Anaplasmataceae* at 20% (25/125) and *Borrelia* spp. at 10.4% (13/125). The less frequent pathogen was *Piroplasmida* at 2.4% (3/125) (Table 2). 

### 3.3. Prevalence of Microorganisms by Sex, Age, Status and Season

In terms of host species, considering only species represented by at least ten individuals, *G. nigeriae* appears to be the most infected species with 59.1% (42/71) of individuals positive for at least one of the microorganisms detected (Table 2). It is also the species with the highest diversity of microorganisms, i.e., four infectious agents out of the five detected (Table 2 and Table 3). However, despite its relatively low abundance, it should be noted that the native *Taterillus* species has a similar profile with the highest diversity of microorganisms including five out of the five detected (Table 3).

**Figure 2:**
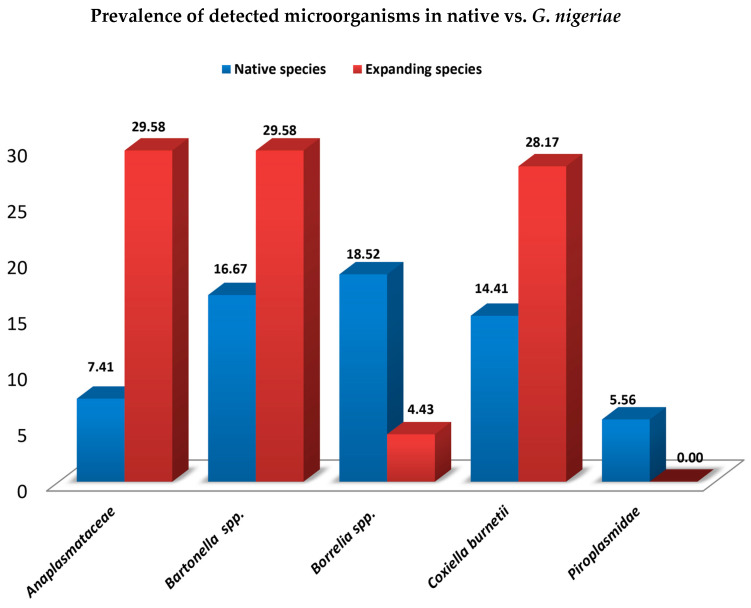
Prevalence of detected microorganisms among 54 native rodents (all native species) in blue and 71 expanding rodents (*G. nigeriae*) in red.

Overall, although the prevalence of microorganisms was higher in *G. nigeriae*, as illustrated in Figure 2, compared to native rodents (all native species), no statistically significant differences were found (W = 20.5, *p*-value = 0.75). There was also no significant difference in the prevalence of microorganisms between males and females (W = 18, *p*-value = 1) or between sampling periods (W = 8, *p*-value = 0.1208). Among the age groups (juveniles vs. adults), however, a statistically significant difference was found, with adults more infected than juveniles (*p*-value = 0.0115; Pearson’s Chi-squared test).

### 3.4. Coinfections with Multiple Microorganisms 

Overall, 19.2% (24/125) of rodents had mixed infections with at least two microorganisms. This included 16 double infections and 8 triple infections. Of these mixed infections, 71% (17/24) was described in *G. nigeriae* compared to 29% (7/24) for all other species (*p*-value *=* 0.4) (Table 2 and Table 3). The association *Anaplasmataceae/C. burnetii* (6/24) represented the most commonly encountered coinfection, followed by *Anaplasmataceae*/*Bartonella/Coxiella* (5/24) and *Anaplasmataceae*/*Bartonella* (4/24).

### 3.5. Phylogenetic Analysis for the Taxonomic Description of Detected Pathogens 

**Figure 3:**
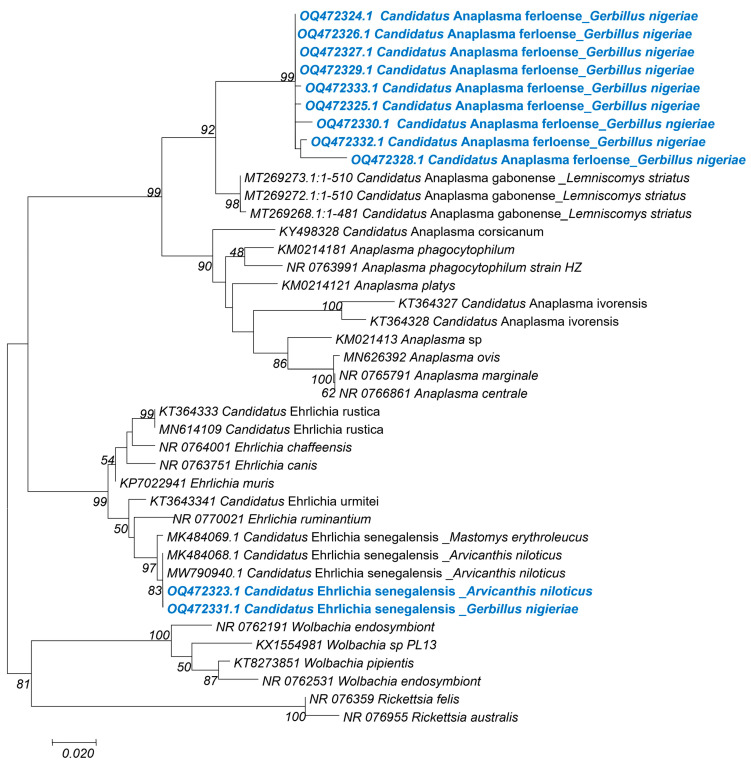
Taxonomic phylogeny of the *Anaplasmatacae* genera and species described in this study. In blue, the sequences of this study. The evolutionary history was inferred by using the Maximum Likelihood method based on the Hasegawa–Kishino–Yano model (HKY). This analysis involved 40 nucleotide sequences. There was a total of 497 positions in the final dataset. Evolutionary analyses were conducted in MEGA7.

*Anaplasmataceae*: A total of 14 23S ribosomal gene sequences, grouped into 2 clusters, were obtained from the 25 *Anaplasmataceae* qPCR-positive samples. The first group consisted of 12 sequences of around 420 bp (OQ472324–OQ472330, OQ472333 and OQ472334), ranging from 99% to 100% identity with one another, and presenting 91% identity with *Anaplasma phagocytophilum* (KM021418) and *Anaplasma platys* (KM021425). These sequences were all obtained from samples of *G. nigeriae* (Figure 3). We consider it to be a putative new species, given its position on the phylogenetic tree and the percentage of homology with the closest valid species. We propose naming it *Candidatus* Anaplasma ferloense. The second group consisted of two sequences (OQ472323 and OQ472331) from *A. niloticus* and *G. nigeriae*, respectively (Figure 3). They were 99% identical to one another and 95% identical to *Ehrlichia ruminantium* (CR92567). However, they also presented 99% homology with *Candidatus* Ehrlichia senegalensis (MK484068 MK484067) and uncultured *Ehrlichia* sp. (OP935909), previously identified in rodents [12] and in *Ornithodoros sonrai* [36] ticks, respectively, both from Senegal.

**Figure 4:**
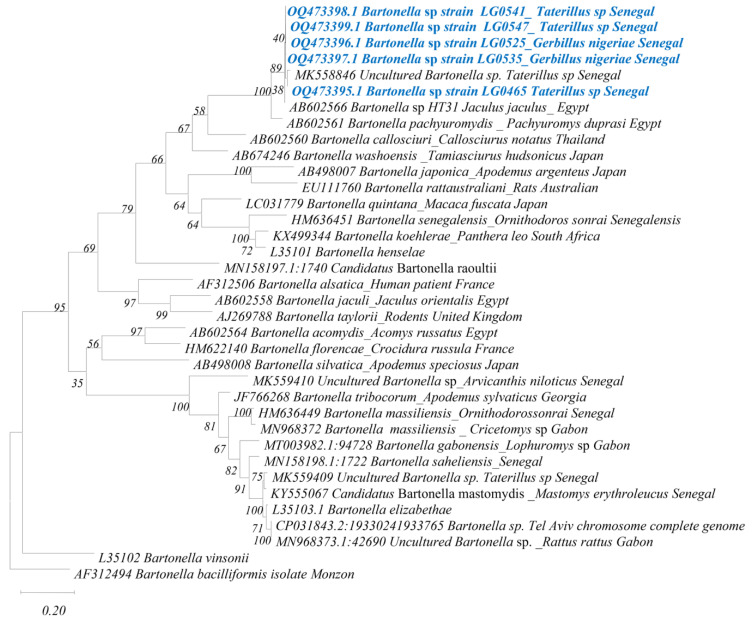
Taxonomic tree and description of the identified *Bartonella* sp. In blue, the sequences of this study and after underscore, the host species of *Bartonella* and the country. The evolutionary history was inferred by using the Maximum Likelihood method and Hasegawa–Kishino–Yano model. This analysis involved 36 nucleotide sequences. There was a total of 610 positions in the final dataset. Evolutionary analyses were conducted in MEGA 7.

*Bartonella* spp.: Among the 30 *Bartonella* qPCR-positive individuals, we obtained 5 sequences with 98–100% identity to one another, including 3 sequences (OQ473395, OQ473398 and OQ473399) from *Taterillus* sp. And 2 sequences (OQ473396 and OQ473397) from *G. nigeriae*. These sequences showed 92% identity with *Bartonella pachyuromydis* (AB602561), described in Egyptian gerbilline rodents *Pachyuromys duprasi* and 98–99% identity with an uncultured *Bartonella* strain found in Senegalese rodents (MK558846). This is probably the same *Bartonella* species (Figure 4).

**Figure 5:**
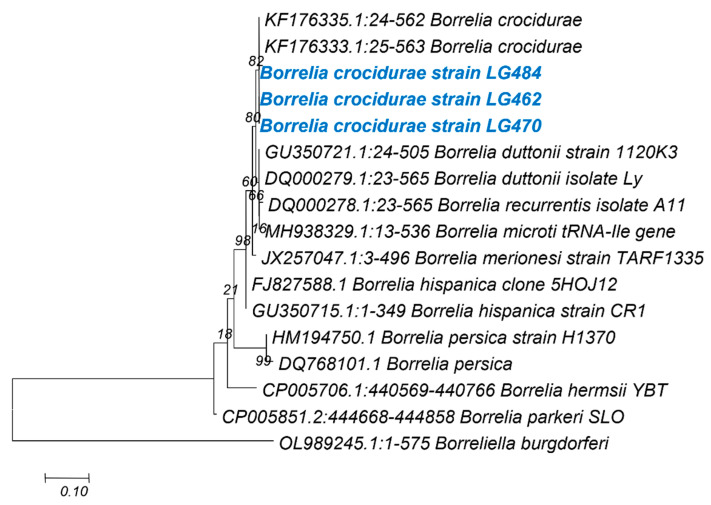
Taxonomic tree and description of the identified *Borrelia* sp. In blue, the sequences of this study. The evolutionary history was inferred by using the Maximum Likelihood method and Hasegawa–Kishino–Yano model. This analysis involved 17 nucleotide sequences. All positions containing gaps and missing data were eliminated. There was a total of 135 positions in the final dataset. Evolutionary analyses were conducted in MEGA7.

***Borrelia* spp.:** A total of 3 (OQ980277-OQ980279) sequences were obtained from the 13 qPCR 16S *Borrelia* sp. Positive samples. Two of these sequences were from *Taterillus* sp. (LG0470 and LG484) and the last one was from *G. nigeriae*. These sequences were identical to one another and showed 99.80% identity with *B. crocidurae* detected in ticks from Mali (JX292946) and 99.63% identity with *B. crocidurae* (KF176340) detected in ticks from Senegal (Figure 5).

## 4. Discussion

We conducted a study based on molecular detection targeting a wide range of microorganisms in the rodents’ spleens. We compared recently arrived and expanding rodents with anciently established indigenous rodents in a region of northern Senegal in order to survey the potential occurrence of rodent-borne zoonotic epidemics. In the Ferlo region, where this investigation is being implemented, a previous study detected and described a diversity of infectious agents in rodents responsible for diseases of concern to public health [12]. This is not the only epidemiological study to have been conducted in Senegalese rodents describing a wide range of microorganisms hosted by these small mammals. Although using different techniques, several studies have revealed the circulation of many zoonotic infectious agents in Senegalese rodents, such as *Toxoplasma gondii* [37,38], *Orthohantavirus*, *Mammarenavirus*, *Orthopoxvirus* [39] and *Flavivirus* [40]. Taken as a whole, the results of these studies justify the need to conduct molecular surveys on the prevalence of microorganisms in small mammals in Senegal, even more so in the context of biological invasion/expansion processes where host–parasite interactions can influence the outcome of the invasion [39].

Furthermore, among the rodents included in this study, we recently highlighted the circulation of a new genotype of *C. burnetii,* which is potentially responsible for an epizootic in these small mammals in the Ferlo. These data, suggesting a high public health risk, were published urgently to alert policy makers to the potential threat that this could represent [21]. This result will, therefore, be included in our discussion.

Our data show the detection of five of the nine microorganisms searched for (Table 1 and Table 2) and the precise identification by sequencing and phylogenetic analysis of three of the microorganisms detected within *Bartonella*, *Anaplasmataceae* and *Borrelia* (Figure 3, Figure 4 and Figure 5); of all the microorganisms detected, these same three infectious agents exhibited the highest prevalence, i.e., 24%, 20% and 10%, respectively.

The *Anaplasmataceae* family consists of numerous genera, including *Anaplasma*, *Aegyptianella, Ehrlichia*, *Neorickettsia*, *Neoehrlichia* and *Wolbachia*. These are gram-negative *Alphaproteobacteria*, small and commonly pleomorphic, which reside in the cytoplasmic vacuoles of host cells [41,42]. Several distinct species of the *Anaplasmataceae* family have been identified as tick-borne human pathogens. Indeed, they infect humans, as well as domestic and wild animals, and are responsible for tick-borne diseases, which are becoming more and more common due to the increase in factors leading to contact between wild animals, their ectoparasites, domestic animals and humans [41]. 

In Senegal, several studies have reported the circulation of *Anaplasmataceae* in domestic animals [24,43] and in rodents [11,12]. Recent data show, for the first time, the detection of *Ehrlichia* sp. in *O. sonrai* ticks, known to be the only vectors of *B. crocidurae*, the agent of tick-borne relapsing fever (TBRF) [36]. Although there are no reported cases of human anaplasmosis, this group of infectious agents continues to receive a great deal of attention both because they affect the economy through disease outbreaks in livestock populations (while also harbouring species known to be pathogenic to humans, such as *A. phagocytophilum*) [43] and because new species are increasingly being identified that could be potentially pathogenic to humans. Our results showed the qPCR detection of *Anaplasmataceae* DNA in 25/125 rodent spleen samples with a prevalence of 20%, comparable to that previously found in the same area (18.8% [12]). *Anaplasmataceae* bacteria were successfully sequenced in 14 samples. The sequences obtained showed the presence of two distinct groups of bacteria. The first group, including 12 samples all from *G. nigeriae,* showed 91% homology with *A. phagocytophilum* (KM021418) and *Anaplasma platys* (KM021425), suggesting that this is a new or undescribed species. This putative new species, named *Candidatus* Anaplasma ferloense, presented 94% to 95% identity with uncultured *Anaplasma* strains known as *Candidatus* Anaplasma gabonense, identified in Gabonese rodents (MT269273) [13]. These two independent, well-supported clusters (*Candidatus* Anaplasma ferloense and *Candidatus* Anaplasma gabonenses) appear as sister groups in a moderately well-supported clade of *Anaplasma* species associated with rodents (Figure 3). The two sequences retrieved from the *A. niloticus* and *G. nigeriae* cluster, with others previously identified in rodents from Senegal and provisionally identified as *Candidatus* Ehrlichia senegalensis (Figure 3), have also been found in *O. sonrai* ticks [36]. This species shows 95% homology with *E. ruminantium* (CR925677). In a study by Dahmana et al. (2020), *Candidatus* Ehrlichia senegalensis was only identified in native rodents, whereas in our study it is also present in expanding *G. nigeriae*, suggesting a possible transfer of infectious agents between native and invasive rodents. This work highlights the identification of potentially new species of *Anaplasmataceae* and emphasises not only the species diversity of this family but also the magnitude of the range of hosts they parasitise.

*Bartonella* species are intracellular, vector-borne, blood-borne gram-negative bacteria that can induce prolonged infection in the host [44]. These infections can be persistent in domestic and wild animals, constituting a significant reservoir of *Bartonella* organisms in nature, which may serve as a source of human infection [44,45]. Indeed, the *Bartonella* genus includes several species that are responsible for zoonotic diseases [46] and are often associated with rodents as their main reservoir hosts [45]. The genus *Bartonella* has been reported several times in both native and invasive rodents in Senegal [11,12]. However, here, we recorded a prevalence of 24% (30/125), much higher than in a previous study by Dahmana et al. 2020: 9% in various sites in the Ferlo region. Kosoy et al. found inter-annual variations in *Bartonella* infection patterns [47]. Additionally, this difference in the prevalence of *Bartonella* infection in sites in the same region could be associated with the period of rodent collection and the diffusion or spread of infection. Indeed, in a study by Dahmana et al., invasive rodents (*G. nigeriae* and *Mus musculus* sp.) collected in 2017 did not show any infection [12], unlike the *G. nigeriae* specimens captured between 2019 and 2020 included in our study. In other African countries, prevalence values of 17.7% [48] and 6.6% [13] have been found in Mali and Gabon, respectively. The high prevalence in our sample rate raises questions about the risk of human infection. Furthermore, of the 30 positive samples, only 5 could be sequenced: 3 from *Taterillus* sp. and 2 from *G. nigeriae*. These five sequences obtained were identical to one another, showing the presence of the same *Bartonella* genotype infecting both species. This genotype showed only 92% identity with *B. pachyuromydis*, which is the closest valid species, indicating, by this low percentage of identity (<98%, according to La Scola et al. 2003 [49]), that it is a new genotype. This genotype has previously been found in Ferlo rodents exclusively in *Taterillus* sp., whereas no detection of *Bartonella* was found in *G. nigeriae* [12].

One endemic species of *Borrelia* in Senegal is *B. crocidurae*, the agent of TBRF in humans [50,51,52]. Borrelia are fastidious bacteria transmitted by ectoparasites (e.g., lice or ticks) and are responsible for various febrile presentations in humans, most often malaria-like symptoms [50]. In Senegal, TBRF caused by *B. crocidurae* is the most common bacterial infection affecting the human population in rural areas. It may be responsible for up to 11% of febrile illnesses recorded in dispensaries [51]. Hosts and reservoirs of this important pathogen have already been studied: previous studies have shown *Crocidura* sp. *A. niloticus*, *Mastomys huberti*, *Mus musculus* sp., *G. nigeriae*, *M. erythroleucus* and *Taterillus* sp. as rodent hosts for TBRF [12,53]. Our study adds *D. braueri* and *G. nancillus* to this list.

In this study, 10% (13/125) of the samples were positive for *Borrelia* spp. through qPCR, and 3 could be sequenced. These sequences were all identified as *B. crocidurae* (KF176340), previously reported in Senegal [11,12] and in other countries of the Sahelo–Saharan region [52,54]. These data show the continuous circulation of *B. crocidurae* and its host variability. It is, therefore, important to continue to search for and identify TBRF in Senegal. In this regard, one recent study reported the detection of *Borrelia* spp. DNA in human skin swabs and dust samples in rural Senegal [55].

Our study revealed the detection of multiple coinfections in rodents, the most represented being *Anaplasmataceae*/*Coxiella* (25%, 6/24), *Anaplasmataceae*/*Bartonella/Coxiella* (21%, 5/24) and *Anaplasmataceae*/*Bartonella* (17%, 4/24) (Table 3). As these infectious agents share the same reservoirs (hosts), it is not surprising to find them in association [56]. In addition, these are infectious agents transmitted by ectoparasites such as ticks, which are known to be vectors of coinfections [57]. However, despite the fact that coinfections are known in rodents [13,57,58], very few studies have reported the *Anaplasma–Bartonella* association [59], and in Senegal there has been no report of it in rodents to date.

## 5. Conclusions

Our study aimed to detect and identify infectious agents harboured by rodents in Ferlo, Senegal. Our results confirm that rodents are hosts of a large number of infectious agents that are potentially pathogenic to humans. Among the six groups of microorganisms found, three were found to present significant prevalence: *Anaplasmataceae*, 20%; *Bartonella*, 24%; and *Borrelia*, 10%. We found no difference in prevalence rates between native rodents and the expanding species *G. nigeriae*, which is currently colonising the region. However, we did observe a potential exchange of bacteria between native species and this recently arrived species. Indeed, potentially new genotypes of *Anaplasmataceae* and *Bartonella* were identified in *G. nigeriae*, whereas in a previous study, they were absent and present only in native rodents. In addition, we found *B. crocidurae*, which is responsible for TBRF and which can have a significant rate of incidence, up to 11% in Senegal. In addition to *Candidatus* Anaplasma ferloense, several undescribed infectious agents have been identified in Senegal in rodents. In order to determine the zoonotic potential of these genotypes and their importance for animal and public health, it would be essential to characterise them.

## Figures and Tables

**Table 1:** Oligonucleotide sequences of primers and probes used for real-time PCR and conventional PCR to detect and identify bacteria and protozoa in this study. For nested PCR: * primers for first PCR, # primers for second PCR.

Target Organism	Target Gene	Technique	Name	SEQUENCES (5′-3′)	Annealing Temperature	Amplicon	Reference
*Anaplasmataceae*	23S	Broad-range qPCR	TtAna_F	TGACAGCGTACCTTTTGCAT	55 °C	190 bp	[24]
			TtAna_R	GTAACAGGTTCGGTCCTCCA			
			TtAna_P	6FAM- GGATTAGACCCGAAACCAAG			
		Broad-range conventional PCR	Ana23S-212F	ATAAGCTGCGGGGAATTGTC	58 °C	960 bp	[24]
			Ana23S-753R	TGCAAAAGGTACGCTGTCAC (for sequencing only)			
			Ana23S-908R	GTAACAGGTTCGGTCCTCCA			
*Bartonella* sp.	ITS (Intergenic 16S–23S)	Broad-range qPCR	Barto_ITS3_F	GATGCCGGGGAAGGTTTTC	60 °C	104 bp	[25]
			Barto_ITS3_R	GCCTGGGAGGACTTGAACCT			
			Barto_ITS3_P	6FAM- GCGCGCGCTTGATAAGCGTG			
		Broad-range conventional PCR	Urbarto1	CTTCGTTTCTCTTTCTTCA	50 °C	733 bp	[26]
			Urbarto2	CTTCTCTTCACAATTTCAAT			
*Borrelia* sp.	16S	Broad-range qPCR	Bor_16S_3F	AGCCTTTAAAGCTTCGCTTGTAG	60 °C	148 bp	[27]
			Bor_16S_3R	GCCTCCCGTAGGAGTCTGG			
			Bor_16S_3P	6FAM- CCGGCCTGAGAGGGTGAACGG			
		Broad-range conventional PCR nested PCR	* Bor_ITS_F	TATGTTTAGTGAGGGGGGTG	56 °C	1034 bp	This study
			* Bor_ITS_R	GATCATAGCTCAGGTGGTTAG			
			# Bor_ITSi_F	GGGGGGTGAAGTCGTAACAAG	60 °C	993 bp	
			# Bor_ITSi_R	TCTGATAAACCTGAGGTCGGA			
*Mycoplasma* sp.	ITS	Broad-range qPCR	Mycop_ITS_F	GGGAGCTGGTAATACCCAAAGT	60 °C	114 bp	[28]
			Mycop_ITS_R	CCATCCCCACGTTCTCGTAG			
			Mycop_ITS_P	6FAM-GCCTAAGGTAGGACTGGTGACTGGGG			
*Rickettsia* sp.	*gltA* (CS)	Broad-range qPCR	RKND03_F	GTGAATGAAAGATTACACTATTTAT	60 °C	166 bp	[25,29]
			RKND03_R	GTATCTTAGCAATCATTCTAATAGC			
			RKND03 P	6-FAM-CTATTATGCTTGCGGCTGTCGGTTC			
*Pan-Filaria*	28S rRNA	Broad-range qPCR	qFil-28S-F	TTG TTT GAG ATT GCA GCC CA	60 °C		[30]
			qFil-28S-R	GTT TCC ATC TCA GCG GTT TC			
			qFil-28S-P	6FAM-CAA GTA CCG TGA GGG AAA GT			
*Piroplasma* sp.	5.8S	Broad-range qPCR	5,8s-F5	AYYKTYAGCGRTGGATGTC	60 °C	40 bp	[31]
			5,8s-R1	TCGCAGRAGTCTKCAAGTC			
			5,8s-S	6-FAM-TTYGCTGCGTCCTTCATCGTTGT			
Pan-*Leishmania/Trypanosoma*	28S LSU	Broad-range qPCR	F Leish/Tryp	AGATCTTGGTTGGCGTAG	60 °C	135 bp	[32]
			R Leish/Tryp	ATAACGTTGTGCTCAGTTTCC			
			P. Leish/Tryp	FAM-GGGAAGGATTTCGTGCCAACG			
Pan-*Kinetoplastidae*	28S LSU (24 alpha)	Broad-range qPCR	F. 24a; 5198	AGTATTGAGCCAAAGAAGG	60 °C	200 bp	[32]
			R. 24a; 5412	TTGTCACGACTTCAGGTTCTAT			
			P. 24a; 5345	FAM- TAGGAAGACCGATAGCGAACAAGTAG			

**Table 2:** Prevalence and diversity of infectious agents detected following rodent species. **# is for the expanding rodent species**.

			Rodent Species							
		Microorganism detected (qPCR-positive individual number)	*A. niloticus* N = 29	*D. braueri* N = 3	*T. pygargus* N = 8	*G. nancillus* N = 9	*J. jaculus* N = 4	**#** *G. nigeriae* N = 71	*X. erythropus* N = 1	**Total** N = 125 (prevalence%)
Prevalence of microorganisms detected		*Anaplasmataceae* (25)	2/29 (6.9%)	0	2/8 (25%)	0	0	21/71 (29.6%)	0	25/125 (20%)
		*Bartonella* spp. (30)	3/29 (10.3%)	1/3 (33.3%)	4/8 (50%)	0	0	21/71 (29.6%)	1/1 (100%)	30/125 (24%)
		*Borrelia* spp. (13)	5/29 (17.2%)	1/3 (33.3%)	3/8 (37.5%)	1/9 (11.1%)	0	3/71 (4.22%)	0	13/125 (10.4%)
		*C. burnetii* (28)	0	1/3 (33.3%)	3/8 (37.5%)	3/9 (33.3%)	1 (25%)	20/71 (28.2%)	0	28/125 (22.4)
		*Piroplasmida* (3)	0	0	3/8 (37.5%)	0	0	0	0	3/125 (2.4%)
Infection type	One infection (43)	*Anaplasma* spp.	1	0	0	0	0	7	0	8
		*Bartonella* spp.	2	1	0	0	0	11	1	15
		*Borrelia* spp.	5	1	1	1	0	1	0	9
		*C. burnetii*	0	0	1	3	1	6	0	11
	Double infections (16)	*Anaplasmataceae/* *Bartonella*	1	0	1	0	0	2	0	4
		Anaplasmataceae/*Borrelia*	0	0	0	0	0	1	0	1
		*Anaplasmataceae/* *Coxiella*	0	0	0	0	0	6	0	6
		*Borrelia/Coxiella*	0	1	0	0	0	0	0	1
		*Bartonella/Coxiella*	0	0	0	0	0	2	0	2
		*Bartonella/Borrelia*	0	0	1	0	0	0	0	1
		*Bartonella/Piroplasmida*	0	0	1	0	0	0	0	1
	Triple infections (8)	*Anaplasmataceae/* *Bartonella/Coxiella*	0	0	0	0	0	5	0	5
		*Anaplasmataceae/* *Coxiella/Piroplasmida*	0	0	1	0	0	0	0	1
		*Bartonella/Borrelia/Coxiella*	0	0	0	0	0	1	0	1
		*Bartonella/Coxiella/Piroplasmida*	0	0	1	0	0	0	0	1
Total infected rodents			9 (31.03%)	3 (100%)	7 (87.5%)	4 (44.4%)	1 (25%)	42 (59.1%)	1 (100%)	67

**Table 3:** Prevalence of infection by age, sex, status and season. 0: not infected; 1: infected.

Label	Variable	Infection		Total	Test
		0	1		
Age	Adults	37 (39.8%)	56 (60.2%)	93 (74.4%)	*p*-value: 0.01
	Juveniles	21 (65.6%)	11 (34.4%)	32 (25.6%)	
	Total	58 (46.4%)	67 (53.6%)	125 (100.0%)	
Sex	Females	32 (51.6%)	30 (48.4%)	62 (49.6%)	*p*-value: 1
	Males	26 (41.3%)	37 (58.7%)	63 (50.4%)	
	Total	58 (46.4%)	67 (53.6%)	125 (100.0%)	
Status	Expanding species	29 (40.8%)	42 (59.2%)	71 (56.8%)	*p*-value: 0.75
	Native species	29 (53.7%)	25 (46.3%)	54 (43.2%)	
	Total	58 (46.4%)	67 (53.6%)	125 (100.0%)	
Season	Dry season	38 (42.2%)	52 (57.8%)	90 (72.0%)	*p*-value: 0.12
	Rainy season	20 (57.1%)	15 (42.9%)	35 (28.0%)	
	Total	58 (46.4%)	67 (53.6%)	125 (100.0%)	

## Data Availability

Not applicable.

## Supplementary Materials

The following supporting information can be downloaded at: https://www.mdpi.com/article/10.3390/genes14051107/s1. Table S1: The list of negative and positive DNA controls used to confirm the sensitivity and specificity of the PCR systems designed for this study.
