# Supplementary material for: Molecular Survey of Rodent-Borne Infectious Agents in the Ferlo Region, Senegal
Source: Genes (Basel). 2023 May 18;14(5):1107. doi: 10.3390/genes14051107 (PMC10218615; doi:10.3390/genes14051107)
Supplement: Supplementary file 1 [file genes-14-01107-s001.zip › genes-2386744-supplementary.pdf]

Table S1. Positive DNA controls used in this study.

Microorganism	Name	Origin
Bacteria	*Anaplasma phagocytophilum*	Laboratory colony IHU
Bacteria	*Bartonella quintana*	Laboratory colony IHU
Bacteria	*Bartonella* sp	Laboratory colony IHU
Bacteria	*Borrelia crocidurae*	Laboratory colony IHU
Bacteria	*Borrrelia* sp	Laboratory colony IHU
Bacteria	*Coxiella burnetii*	Laboratory colony IHU
Bacteria	*Rickettsia australis*	Laboratory colony IHU
Bacteria	*Rickettsia canadensis*	Laboratory colony IHU
Bacteria	*Rickettsia conorii*	Laboratory colony IHU
Bacteria	*Rickettsia felis*	Laboratory colony IHU
Bacteria	*Streptobacillus moniliformis*	Laboratory colony IHU
Bacteria	*Wolbachia*	Laboratory colony IHU
Bacteria	*Yersinia pestis*	Laboratory colony IHU
Parasite	*Leishmania*	Laboratory colony IHU
Parasite	*Leishmania major*	Laboratory colony IHU
Parasite	*Trypanosoma congolense*	Laboratory colony IHU
Parasite	*Trypanosoma gambiense*	Laboratory colony IHU
Parasite	*Toxoplasma gondi*	Positive patient DNA, IHU laboratory
Parasite	*Piroplasms*	DNA from positive rodents for piroplasms [1]
